# Pharmacological insights into the antidiabetic potential of *Rosa hybrida* Hort. flowers based on enzyme inhibition and computational analysis

**DOI:** 10.1016/j.toxrep.2026.102334

**Published:** 2026-08-25

**Authors:** Entris Sutrisno, Risa Susanti, Jajang Japar Sodik, Nabila Hadiah Akbar, Taufik Muhammad Fakih, Salsabila Adlina, Saeful Amin

**Affiliations:** a Department of Pharmacology and Clinical Pharmacy, Faculty of Pharmacy, Universitas Bhakti Kencana, Jl. Soekarno-Hatta No. 754, Bandung, 40614, Indonesia; b Pharmacist Professional Education Study Program (PPSP), Faculty of Mathematics and Natural Sciences, Universitas Lambung Mangkurat, Jl. Jenderal Ahmad Yani Km 36, Banjarbaru, 70714, Indonesia; c Department of Pharmaceutical Analysis and Medicinal Chemistry, Faculty of Pharmacy, Universitas Bhakti Kencana, Jl. Soekarno-Hatta No. 754, Bandung, 40614, Indonesia; d Laboratorium Terpadu, Universitas Lambung Mangkurat, Jl. Unlam I, Banjarbaru, 70714, Indonesia; e Department of Pharmacy, Faculty of Mathematics and Natural Sciences, Universitas Islam Bandung, Jl. Ranggagading No. 8, Bandung, 40116, Indonesia; f Department of Pharmacy, Faculty of Health Sciences, Universitas Perjuangan Tasikmalaya, Jl. Peta No. 177, Tasikmalaya, 46115, Indonesia; g Department of Pharmacy, Faculty of Pharmacy, Universitas Bakti Tunas Husada, Jl. Letjen Mashudi No. 20, Tasikmalaya, 46196, Indonesia

**Keywords:** *Rosa hybrida* Hort, Antidiabetic activity, α-Glucosidase and α-Amylase inhibition, Antioxidant activity, Molecular docking

## Abstract

*Rosa hybrida* Hort. flowers are widely recognized for their ornamental value; however, their pharmacological potential, particularly as natural antidiabetic agents, remains insufficiently explored. This study aimed to evaluate the antioxidant and antidiabetic potential of *Rosa hybrida* Hort. flower extract using integrated *in vitro* and *in silico* approaches. The flower extract was prepared by ethanolic maceration following botanical authentication. Antioxidant activity was assessed using the DPPH radical scavenging assay, while total phenolic content (TPC) and total flavonoid content (TFC) were determined using the Folin–Ciocalteu and aluminum chloride colorimetric methods, respectively. Antidiabetic activity was evaluated through α-glucosidase and α-amylase inhibitory assays, with acarbose as a positive control. Metabolite profiling was conducted using LC–MS/MS, and selected bioactive compounds were further investigated by molecular docking to elucidate interactions with target enzymes. The extract exhibited very strong antioxidant activity, with an IC₅₀ value of 9.78 µg/mL, supported by high TPC (60.51 mg GAE/100 mg extract) and TFC (38.51 mg QE/100 mg extract). Potent α-glucosidase inhibitory activity was observed (IC₅₀ = 4.45 ppm), while inhibition of α-amylase was moderate to weak (IC₅₀ = 17,149.28 ppm), indicating favorable enzyme selectivity. LC–MS/MS analysis identified major phenolic constituents, including quercetin, kaempferol, hyperoside, cyanidin-3-O-arabinopyranoside, and tricoumaroyl spermidine, associated with antioxidant and antidiabetic activities. Molecular docking supported these findings by revealing stable interactions between selected metabolites and catalytic residues of α-glucosidase and α-amylase. *Rosa hybrida* Hort. flower extract exhibits significant antioxidant and antidiabetic potential, primarily through selective α-glucosidase inhibition, highlighting its promise as a natural source for functional antidiabetic agent development.

## Highlights

• *Rosa hybrida* extract showed strong DPPH activity with an IC₅₀ of 9.78 µg/mL.
• TPC and TFC reached 60.51 mg GAE and 38.51 mg QE per 100 mg extract.
• α-Glucosidase inhibition (IC₅₀ 4.45 ppm) greatly exceeded α-amylase inhibition.
• LC–MS/MS identified quercetin, kaempferol, hyperoside, and other metabolites.
• Docking revealed metabolite interactions with α-glucosidase and α-amylase residues.

## Graphical Abstract

**Figure:**
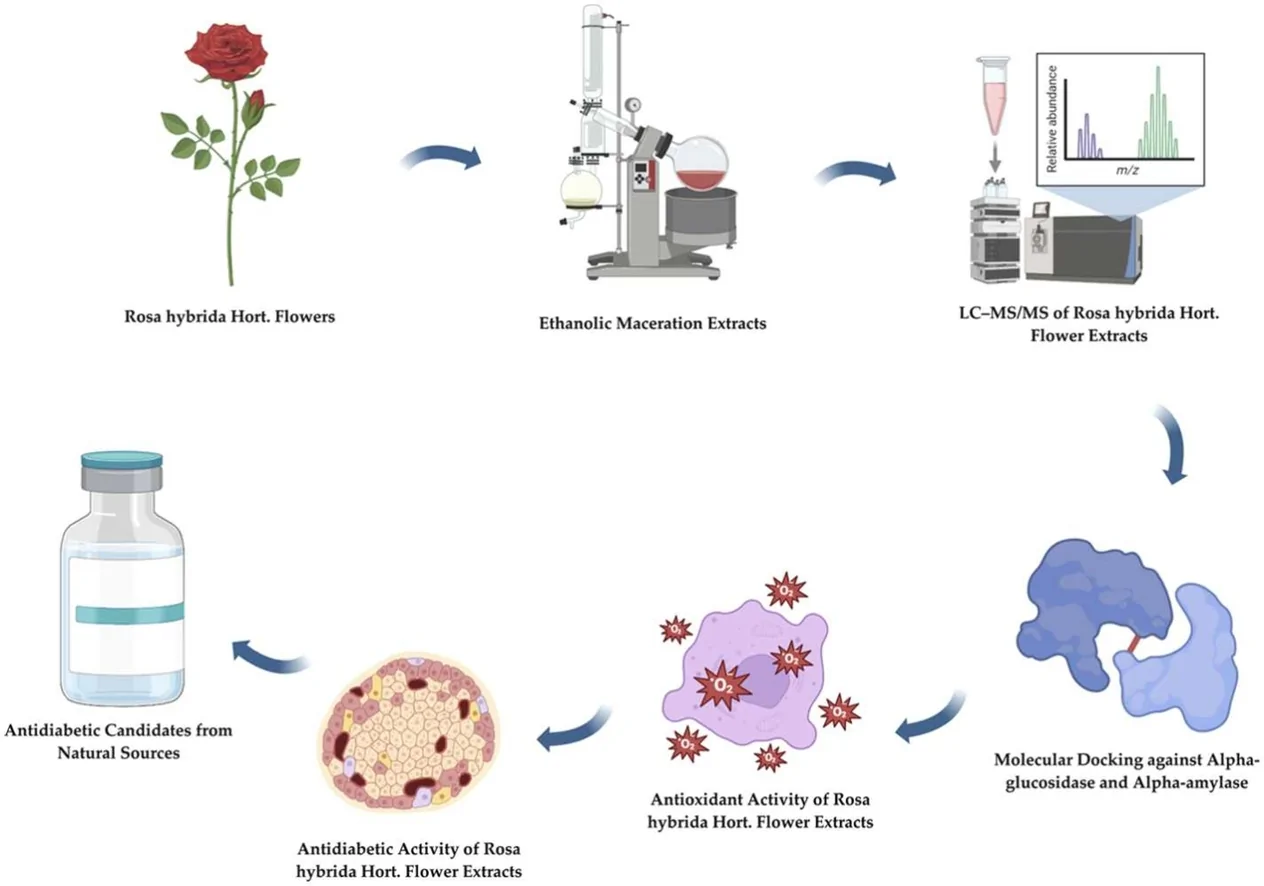

## 1 Introduction

Diabetes mellitus (DM) is a global health problem with a prevalence that continues to increase worldwide. According to data from the International Diabetes Federation (IDF) in 2021, approximately 537 million adults aged 20–79 years were living with diabetes, representing 10.5% of the population in this age group [1]. This number is projected to increase to 783 million by 2045 due to population growth, urbanization, and lifestyle changes [2]. Diabetes mellitus is classified as one of the most serious metabolic disorders affecting global health systems. More than 90% of diabetes cases are categorized as type 2 diabetes mellitus (T2DM), which is strongly associated with insulin resistance and impaired glucose metabolism [3]. Persistent hyperglycemia in T2DM leads to progressive metabolic dysregulation. If not properly managed, T2DM can result in severe complications such as cardiovascular disease, nephropathy, neuropathy, and retinopathy [4]. These complications significantly increase morbidity and mortality rates among diabetic patients. Effective glycemic control is therefore essential for preventing long-term complications. Although various antidiabetic drugs are currently available, many patients still fail to achieve optimal blood glucose control. Therapeutic regimens often require long-term administration and strict patient adherence [5]. Common antidiabetic medications, including insulin, sulfonylureas, and α-glucosidase inhibitors such as acarbose, are widely prescribed. These agents effectively reduce blood glucose levels through different mechanisms of action. However, their use is frequently associated with adverse effects [6]. Such limitations highlight the need for alternative therapeutic strategies for diabetes management.

Conventional antidiabetic therapies are often linked to side effects such as hypoglycemia, weight gain, and gastrointestinal disturbances. These adverse effects may reduce patient compliance and limit long-term treatment efficacy [7]. In addition, the high cost of some antidiabetic medications presents a financial burden, particularly in low- and middle-income countries. These challenges have encouraged the exploration of alternative therapeutic approaches that are safer, more affordable, and more accessible [8]. Herbal medicine has long been utilized in traditional systems for the management of diabetes. In recent years, interest in herbal antidiabetic therapies has increased due to their favorable safety profiles [9]. Medicinal plants are known to contain diverse bioactive compounds with potential antidiabetic effects. These compounds include polyphenols, such as flavonoids, tannins, and anthocyanins, as well as terpenoids and phytosterols. Polyphenolic compounds are particularly recognized for their antioxidant properties [10], [11]. Oxidative stress plays a critical role in the pathogenesis of diabetes and its complications. Therefore, antioxidants may contribute to glycemic control by reducing oxidative damage [12]. Plant-derived compounds can also improve insulin sensitivity and modulate glucose metabolism. Another important mechanism involves the inhibition of carbohydrate-digesting enzymes. Enzymes such as α-glucosidase and α-amylase are responsible for glucose release from dietary carbohydrates [13]. Inhibiting these enzymes can effectively reduce postprandial hyperglycemia.

Beyond their antioxidant properties, natural bioactive compounds have attracted considerable attention because of their pleiotropic pharmacological activities, enabling them to modulate multiple biological pathways involved in the development and progression of chronic diseases [14], [15]. Polyphenols, flavonoids, phenolic acids, alkaloids, terpenoids, and other phytochemicals have been reported to exert anti-inflammatory, antioxidant, anti-obesity, cardioprotective, neuroprotective, antimicrobial, and anticancer activities through the regulation of oxidative stress, inflammatory signaling, mitochondrial function, and cellular metabolism [16]. Increasing evidence also suggests that these compounds can simultaneously influence several molecular targets associated with glucose homeostasis, including insulin signaling, glucose transport, pancreatic β-cell function, lipid metabolism, and digestive enzymes, thereby providing multitarget therapeutic benefits for diabetes management [17]. In addition, many plant-derived metabolites exhibit favorable safety profiles and may complement conventional pharmacotherapy while reducing the risk of adverse effects associated with long-term synthetic drug use [18]. These multifunctional biological properties have stimulated extensive research into medicinal plants as valuable sources of novel bioactive compounds for the prevention and treatment of metabolic disorders, including T2DM [13]. Therefore, investigating medicinal plants with diverse phytochemical compositions represents a rational strategy for identifying new natural antidiabetic agents with multiple complementary mechanisms of action.

Among carbohydrate-digesting enzymes, α-glucosidase inhibition is considered a particularly effective strategy for managing T2DM. Inhibition of α-glucosidase delays the breakdown of disaccharides into absorbable monosaccharides. This delay reduces the rate of glucose absorption in the small intestine [19]. As a result, postprandial blood glucose spikes can be significantly attenuated. Several synthetic α-glucosidase inhibitors are currently used in clinical practice [20]. However, these drugs are often associated with gastrointestinal side effects. Consequently, there is growing interest in identifying natural α-glucosidase inhibitors from plant sources [21]. Numerous medicinal plants have been reported to exhibit α-glucosidase inhibitory activity. Natural inhibitors are generally considered to have better tolerability profiles. In addition to enzyme inhibition, many plant extracts exhibit antioxidant activity [22]. The combination of antioxidant and enzyme inhibitory effects enhances their therapeutic potential. One promising natural resource for antidiabetic development is *Rosa hybrida* Hort., commonly known as the hybrid rose. Roses are widely cultivated as ornamental plants throughout the world. Beyond their aesthetic value, roses possess a variety of pharmacological properties [23], [24]. Traditional medicine has utilized different parts of the rose plant for therapeutic purposes.

Various parts of the rose plant, including flowers, fruits, leaves, and bark, have been traditionally used in herbal remedies. Previous studies have reported that rose extracts exhibit anti-inflammatory, antidepressant, antibacterial, and antidiabetic activities [25]. The medicinal properties of roses are largely attributed to their rich phytochemical composition. In particular, rose flowers are abundant in phenolic compounds and flavonoids [26]. These secondary metabolites are known to contribute to antioxidant and enzyme inhibitory activities. Recent studies have demonstrated that *Rosa hybrida* flower extracts with intense pigmentation contain higher concentrations of phenolic and flavonoid compounds [27]. Such extracts have shown strong antioxidant activity in DPPH radical scavenging assays. The antioxidant potential of these extracts suggests their ability to counteract oxidative stress associated with diabetes [28]. Moreover, several studies have reported potent α-glucosidase inhibitory activity of *Rosa hybrida* flower extracts. Interestingly, the inhibitory effect against α-glucosidase is often stronger than that of standard synthetic inhibitors such as acarbose [29]. In contrast, the inhibition of α-amylase by rose extracts tends to be moderate. This selective enzyme inhibition is considered advantageous. Selective α-glucosidase inhibition minimizes excessive suppression of α-amylase activity [30]. Therefore, *Rosa hybrida* flowers represent a promising source of natural antidiabetic agents.

Based on these considerations, this study aims to comprehensively investigate the antidiabetic potential of *Rosa hybrida* flower extract. The first objective is to evaluate the antioxidant activity of the extract using the DPPH radical scavenging assay. In addition, total phenolic content and total flavonoid content are determined to support antioxidant findings. The second objective is to assess the antidiabetic activity of the extract through α-glucosidase and α-amylase inhibition assays. These assays provide insight into the extract’s ability to modulate carbohydrate digestion. The third objective is to identify the bioactive compounds present in the extract using LC–MS/MS analysis. Metabolite profiling allows for the characterization of compounds potentially responsible for the observed activities. The fourth objective is to investigate the antidiabetic potential of the identified compounds through molecular docking studies. Docking simulations are performed against α-glucosidase and α-amylase enzyme receptors. This *in silico* approach provides molecular-level insight into ligand–enzyme interactions. The integration of *in vitro* and *in silico* methods strengthens the mechanistic interpretation of the results. This combined strategy allows for a more comprehensive understanding of antidiabetic activity. The findings of this study are expected to provide scientific evidence supporting the use of *Rosa hybrida* as an antidiabetic herbal resource. Ultimately, this research may contribute to the development of novel natural antidiabetic agents and pharmaceutical innovations.

## 2 Materials and methods

### 2.1 Plant collection and identification

Fresh *Rosa hybrida* Hort. flowers, locally known as mawar merah, were collected from Kebun Mawar Parongpong, Lembang, West Java, Indonesia. The collection site is recognized for the cultivation of ornamental rose varieties under controlled horticultural conditions. Plant material was harvested during the flowering stage to ensure optimal phytochemical content. The collected specimens were immediately transported to the laboratory for further processing. Botanical authentication was performed at the Herbarium Jatinagoriense, Laboratory of Biosystematics and Molecular Biology, Department of Biology, Faculty of Mathematics and Natural Sciences, Universitas Padjadjaran, Jatinangor, West Java, Indonesia. Taxonomic identification was conducted using macroscopic and microscopic morphological characteristics. The specimen was officially confirmed as *Rosa hybrida* Hort., belonging to the *Rosaceae* family. The authentication process was documented under Plant Identification Sheet No. 276/LBM/IT/XII/2023.

### 2.2 Extract preparation

Authenticated *Rosa hybrida* Hort. petals were separated from other floral parts prior to processing. The petals were air-dried under shaded conditions to prevent degradation of thermolabile constituents. Drying was continued until a constant weight was achieved. The dried material was pulverized using a mechanical grinder to obtain a uniform powder with a particle size of 40–60 mesh. A total of 100 g of powdered petals was used for extraction. Maceration was performed using 95% ethanol as the extraction solvent. The extraction was conducted at room temperature (25–28 °C) for 3 × 24 h with occasional stirring. After each 24-hour cycle, the mixture was filtered to separate the filtrate from the plant residue. The remaining plant residue was re-macerated twice using fresh ethanol to ensure exhaustive extraction. All filtrates were combined into a single extract solution. The pooled filtrate was concentrated under reduced pressure using a rotary evaporator at 45 ± 2 °C. Concentration continued until a viscous crude extract was obtained. The extract was transferred into amber-colored glass bottles to protect it from light exposure. The samples were stored at 4 °C prior to further analysis [31].

### 2.3 Antioxidant activity assay

Antioxidant activity was evaluated using the 2,2-diphenyl-1-picrylhydrazyl (DPPH) radical scavenging assay. The method was adapted from Blois with minor modifications. Various concentrations of the extract were prepared in methanol. Each sample solution was mixed with DPPH solution (50 µg/mL) at a 1:1 vol ratio. Methanol with DPPH served as the control solution. Ascorbic acid was used as the reference standard. The reaction mixtures were incubated in the dark at room temperature for 30 min. Absorbance was measured at 516 nm using a UV–Vis spectrophotometer (Shimadzu UV-1800). All measurements were conducted in triplicate. Radical scavenging activity was calculated as a percentage of inhibition. The calculation was based on the difference between control and sample absorbance values. The IC₅₀ value was defined as the concentration required to inhibit 50% of DPPH radicals. IC₅₀ values were obtained from a calibration curve plotting concentration against inhibition percentage. This assay provides a quantitative measure of antioxidant capacity. The results reflect the hydrogen-donating ability of the extract. Antioxidant activity data were used to support antidiabetic evaluations [32].

### 2.4 Total phenolic content

Total phenolic content was determined using the Folin–Ciocalteu colorimetric method with slight modifications. A 0.5 mL aliquot of each extract was mixed with 5 mL of 10% Folin–Ciocalteu reagent. The mixture was allowed to react for a short pre-incubation period. Subsequently, 4 mL of 1 M sodium carbonate solution was added. The reaction mixture was incubated at room temperature for 15 min. During incubation, a blue-colored complex was formed. Absorbance was measured at 765 nm using a UV–Vis spectrophotometer. Each sample was analyzed in triplicate to ensure reproducibility. Gallic acid was used as the reference standard. A calibration curve was prepared using gallic acid concentrations ranging from 40 to 100 µg/mL. Total phenolic content was calculated based on the calibration curve. Results were expressed as grams of gallic acid equivalents per 100 g of extract. The Folin–Ciocalteu method provides an estimate of total reducing capacity. Phenolic content is closely associated with antioxidant potential. These data were used to support the bioactivity profile of the extract [33].

### 2.5 Total flavonoid content

Total flavonoid content was determined using a modified aluminum chloride colorimetric method. A 0.5 mL aliquot of each extract was mixed with 0.1 mL of 10% aluminum chloride solution. Subsequently, 0.1 mL of 1 M sodium acetate was added to the mixture. Distilled water (2.8 mL) was then added to achieve the final reaction volume. The mixture was further diluted with 1.5 mL of ethanol. The reaction mixture was incubated at room temperature for 15 min. Formation of a yellow-colored complex indicated flavonoid–aluminum interactions. Absorbance was measured at 415 nm using a UV–Vis spectrophotometer. All analyses were performed in triplicate. Quercetin was used as the standard compound. A calibration curve was prepared using quercetin concentrations ranging from 50 to 100 µg/mL. Total flavonoid content was calculated from the calibration curve. Results were expressed as grams of quercetin equivalents per 100 g of extract. This assay estimates the total flavonoid concentration in the sample. Flavonoids are known contributors to antioxidant and enzyme inhibitory activities. The obtained values support the phytochemical richness of the extract [34].

### 2.6 Alpha-glucosidase inhibitory activity

Alpha-glucosidase inhibitory activity was evaluated using a modified standard protocol. The assay was conducted in a 96-well microplate format. Each well contained 50 µL of phosphate buffer (100 mM, pH 6.8). Alpha-glucosidase enzyme solution (10 µL, 1 U/mL) was added to each well. Sample extract or acarbose standard (20 µL) at various concentrations was then introduced. The reaction mixture was incubated at 37 °C for 15 min. Following incubation, 20 µL of 5 mM p-nitrophenyl-β-D-glucopyranoside substrate was added. The mixture was incubated again at 37 °C for 20 min. The enzymatic reaction was terminated by adding 50 µL of 0.1 M sodium carbonate. Enzyme activity was determined based on p-nitrophenol release. Absorbance was measured at 405 nm using a Multiskan microplate reader. All experiments were conducted in triplicate. Percentage inhibition was calculated relative to the control. The control contained enzyme and buffer without inhibitor. IC₅₀ values were calculated from inhibition curves. This assay evaluates the ability of the extract to inhibit postprandial glucose release [35].

### 2.7 Alpha-amylase inhibitory activity

Alpha-amylase inhibitory activity was assessed using a modified colorimetric method. The assay was performed in a 96-well microplate. Each well contained 50 µL of phosphate buffer (100 mM, pH 6.8). Alpha-amylase enzyme solution (10 µL, 2 U/mL) was added to the buffer. Sample extract or standard at various concentrations was then introduced. The reaction mixture was incubated at 37 °C for 20 min. Subsequently, 10 µL of 1% soluble starch solution was added as the substrate. The mixture was incubated for an additional 30 min at 37 °C. DNS reagent (100 µL) was then added to stop the reaction. The plate was heated at 95 °C for 15 min to develop color. After cooling, absorbance was measured at 450 nm using a microplate reader. All experiments were conducted in triplicate. Percentage inhibition was calculated using the same formula as for the α-glucosidase assay. Acarbose served as the positive control. This assay evaluates starch hydrolysis inhibition. Moderate α-amylase inhibition is considered advantageous for antidiabetic therapy [36].

### 2.8 LC–MS/MS profiling of *Rosa hybrida* Hort. extract

LC–MS/MS analysis was performed using an Agilent 1290 Infinity II UHPLC system coupled with a 6545 Q-TOF mass spectrometer. Chromatographic separation was achieved using a ZORBAX Eclipse Plus C18 column. The column dimensions were 100 mm × 2.1 mm with a particle size of 1.8 µm. The column temperature was maintained at 35 °C. The mobile phase consisted of solvent A (0.1% formic acid in water) and solvent B (0.1% formic acid in acetonitrile). A gradient elution program was applied over 22 min. The flow rate was set at 0.3 mL/min. The injection volume was 2 µL. The mass spectrometer operated in both positive and negative electrospray ionization modes. Source parameters were optimized to ensure stable ionization. Data acquisition was performed in full-scan mode over an *m/z* range of 100–1000. Auto MS/MS acquisition was conducted using collision energies of 20–40 eV. Internal reference ions were used for continuous mass calibration. Metabolite identification was achieved by comparison with spectral databases. This analysis enabled tentative identification of major bioactive compounds [37].

### 2.9 Molecular docking validation

Molecular docking validation was conducted using AutoDock version 4.2.3 [38]. The native ligand was first separated from the target protein using Discovery Studio Visualizer 2025 [39]. Redocking of the native ligand was performed to validate the docking protocol. The grid center was positioned at the original binding site of the native ligand. Gridbox dimensions were adjusted to include all key binding residues. Docking simulations were performed using the Lamarckian Genetic Algorithm. A total of 100 genetic algorithm runs were executed. Medium evaluation parameters were applied to balance accuracy and computational efficiency. The resulting docked conformations were compared with the crystallographic pose. Root Mean Square Deviation values were calculated for validation. A docking protocol was considered valid if the RMSD value was ≤ 2 Å [40]. This criterion indicates accurate reproduction of the native binding mode. Validation ensures reliability of subsequent docking simulations. Proper validation reduces the risk of false-positive predictions. The validated protocol was used for ligand docking studies. This step is essential for computational accuracy.

### 2.10 Molecular docking simulation

Molecular docking simulations were performed using PyRx version 0.9.2 with the AutoDock4 algorithm [41]. Test ligands identified from LC–MS/MS analysis were prepared prior to docking. Protein preparation included removal of water molecules and addition of polar hydrogens. Gridbox parameters were set based on validation results. Docking simulations were executed for each ligand independently. Multiple docking poses were generated for each ligand. Binding affinity was evaluated based on ΔG values. The most stable conformation was selected based on lowest binding energy. Intermolecular interactions were analyzed for selected poses. Hydrogen bonding, hydrophobic interactions, and electrostatic contacts were evaluated. Docking results were compared across ligands. The binding modes provided insights into enzyme inhibition mechanisms. This simulation complements *in vitro* inhibition data. Docking analysis supports structure–activity relationships. Computational results enhance mechanistic interpretation. The docking approach strengthens the overall antidiabetic evaluation.

### 2.11 Molecular docking visualization

Docking visualization was conducted to analyze ligand–protein interactions in detail. The PLIP (Protein–Ligand Interaction Profiler) version 2.3.1 was used to identify interaction types [42]. Discovery Studio Visualizer 2025 was employed for two-dimensional interaction mapping. Three-dimensional visualization was performed using Visual Molecular Dynamics (VMD) [43]. These tools enabled comprehensive interpretation of docking results. Hydrogen bonds, hydrophobic interactions, and π-interactions were identified. Interaction distances and involved amino acid residues were analyzed. Visualization aided in understanding binding orientation. Comparative analysis was performed among different ligands. Interaction profiles were correlated with binding affinity values. Visualization results supported docking score interpretations. These analyses helped identify key residues involved in enzyme inhibition. Graphical representations improved clarity of molecular interactions. Visualization strengthens structure–function interpretation. The combined tools provided complementary insights. This approach enhances the reliability of docking conclusions.

## 3 Results

### 3.1 Plant collection and identification

Accurate botanical identification is a critical step in natural product research, as misidentification can lead to erroneous interpretation of biological activities and compromise the reproducibility of research findings. In this study, red rose flowers (*Rosa hybrida* Hort.) were collected from Parongpong District, West Bandung Regency, West Java Province, Indonesia, and were taxonomically authenticated at the Herbarium Jatinagoriense, Plant Taxonomy Laboratory, Department of Biology, Faculty of Mathematics and Natural Sciences, Universitas Padjadjaran. The results of the taxonomic determination confirmed that the plant specimen used in this study was *Rosa hybrida* Hort., belonging to the family *Rosaceae*, as documented in Plant Identification Sheet No. 276/LBM/IT/XII/2023. The availability of this authentication record strengthens the scientific validity of the study and ensures the traceability of the plant material employed. International pharmacognosy research guidelines emphasize that the deposition of a voucher specimen is an essential requirement for ensuring the credibility, transparency, and reproducibility of natural product research. The family *Rosaceae*, particularly the genus *Rosa*, is known to be rich in secondary metabolites such as flavonoids, phenolic compounds, and anthocyanins, which exhibit antioxidant and antidiabetic activities. However, phytochemical composition and biological activity may vary significantly depending on species, cultivar, environmental conditions, and geographical origin. Therefore, rigorous botanical authentication is a fundamental prerequisite to ensure that the observed biological activities are accurately attributed to the plant species under investigation.

### 3.2 Sample preparation and extraction process

The preparation of plant material involved wet sorting, washing, cutting, and oven drying at 50 °C prior to grinding into simplicia powder. Drying at this moderate temperature was intended to reduce moisture content while minimizing the degradation of thermolabile active compounds, particularly phenolic compounds and flavonoids, which are sensitive to elevated temperatures and oxidative conditions. Extraction of red rose flowers was performed using the maceration method with 96% ethanol as the solvent. Maceration was selected because it does not involve direct heating, thereby preserving the stability of heat-sensitive bioactive compounds and preventing disruption of cell wall structures that could reduce the yield of secondary metabolites. In addition, this method is relatively simple, cost-effective, and widely applied in phytochemical and pharmacological studies of natural products. The use of ethanol as an extraction solvent offers several advantages, including its ability to dissolve polar to semi-nonpolar compounds such as flavonoids, tannins, alkaloids, and anthocyanins. Ethanol is also considered safer and less toxic than many other organic solvents, making it suitable for extracts intended for biological evaluation. Furthermore, ethanol exhibits lower susceptibility to microbial contamination, which enhances extract stability during processing and storage. Concentration of the extract was carried out using a rotary evaporator under reduced pressure and controlled temperature, allowing solvent removal at temperatures below its normal boiling point. This approach was employed to preserve the stability of bioactive compounds and prevent thermal degradation during concentration. The resulting extract was subsequently stored at 4 °C in sealed, dark-colored containers to minimize degradation caused by light exposure and oxidation. Such storage conditions are particularly important for protecting phenolic compounds and anthocyanins, which are known to be photosensitive and prone to oxidative degradation.

### 3.3 Phytochemical screening

The results of phytochemical screening, as presented in Table 1, revealed that the simplicia of red rose flowers (*Rosa hybrida* Hort.) contains a wide range of secondary metabolite classes, including alkaloids, flavonoids, tannins, quinones, saponins, steroids/triterpenoids, and anthocyanins. The presence of multiple metabolite classes indicates a chemically complex phytochemical profile. Such complexity is advantageous in natural product research because different compound classes may contribute synergistically to biological activity. Qualitative phytochemical screening serves as an important preliminary step to predict potential pharmacological properties. The detected flavonoids, tannins, and anthocyanins belong to the phenolic compound group, which is widely recognized for its strong antioxidant capacity. These compounds exert antioxidant effects through free radical scavenging, hydrogen or electron donation, and chelation of metal ions involved in oxidative reactions. Oxidative stress plays a central role in the pathogenesis of diabetes mellitus, particularly in pancreatic β-cell dysfunction and insulin resistance. Excessive reactive oxygen species can impair insulin signaling and exacerbate hyperglycemic conditions. Therefore, the presence of phenolic compounds in *Rosa hybrida* flower extract is highly relevant for antidiabetic applications. Phenolic-rich extracts are frequently associated with improved antioxidant defense mechanisms. This antioxidant activity may help protect pancreatic tissue from oxidative damage. In addition, antioxidant compounds can indirectly improve insulin sensitivity. The detection of these metabolites provides a scientific basis for evaluating antioxidant activity using quantitative assays. These findings also support further investigation of enzyme inhibitory activity. The phytochemical profile suggests that the extract has promising therapeutic potential.

**Table 1:** Phytochemical Screening Results of *Rosa hybrida* Hort. Flower Extract.

**No**	**Classes of Compounds**	**Results**
1	Alkaloids	+
2	Flavonoids	+
3	Tannins	+
4	Quinones	+
5	Saponins	+
6	Steroids and Triterpenoids	+
7	Anthocyanins	+

The detection of saponins and steroids/triterpenoids further supports the antidiabetic potential of red rose flower extract. These compound classes have been reported to inhibit carbohydrate-digesting enzymes such as α-glucosidase and α-amylase. Inhibition of these enzymes delays carbohydrate hydrolysis and glucose absorption in the small intestine. This mechanism effectively reduces postprandial blood glucose levels, which is a key therapeutic target in type 2 diabetes mellitus. Selective inhibition of α-glucosidase is considered particularly beneficial to minimize gastrointestinal side effects. Triterpenoids have also been reported to modulate insulin sensitivity and glucose uptake at the cellular level. Saponins may additionally influence lipid metabolism and contribute to improved glycemic control. Alkaloids identified in the extract are known to interact with enzyme active sites and regulate signaling pathways related to glucose metabolism. Some alkaloids exhibit insulin-mimetic or insulin-secretagogue properties. Quinones are redox-active compounds that can modulate oxidative stress pathways and cellular energy metabolism. Their presence may enhance both antioxidant and antidiabetic effects of the extract. Anthocyanins, which are responsible for the red pigmentation of rose petals, are well known for their antioxidant and anti-inflammatory activities. These compounds have been reported to improve glucose metabolism and protect pancreatic β-cells from oxidative damage. The coexistence of multiple bioactive compound classes suggests the possibility of synergistic interactions. Such interactions are often considered a key advantage of herbal extracts over single-compound therapies.

### 3.4 Antioxidant activity assay, total phenolic content, and total flavonoid content

The antioxidant activity of the ethanolic extract of *Rosa hybrida* Hort. flowers was evaluated using the DPPH radical scavenging assay, with the IC₅₀ value serving as the main indicator of antioxidant potency. The DPPH radical is a stable nitrogen-centered free radical characterized by a deep purple color that becomes pale yellow upon reduction by antioxidant compounds through electron or hydrogen atom donation. As shown in Table 2, lower IC₅₀ values indicate stronger antioxidant capacity, as they reflect a lower concentration required to neutralize 50% of DPPH radicals. In this study, the extract exhibited an IC₅₀ value of 9.775 ± 0.025 µg/mL, while vitamin C, used as a positive control, showed an IC₅₀ value of 3.909 ± 0.019 µg/mL. The low IC₅₀ value of vitamin C confirms its role as a highly potent antioxidant reference. Based on commonly accepted criteria, antioxidant activity is classified as very strong when IC₅₀ values are below 50 µg/mL. Accordingly, the *Rosa hybrida* flower extract can be categorized as a very strong antioxidant. Although the extract exhibited lower potency than pure vitamin C, its antioxidant strength remains remarkable for a crude plant extract. This finding indicates that the extract contains compounds capable of efficiently scavenging free radicals. The strong DPPH scavenging activity suggests effective electron- or hydrogen-donating ability. Such activity is relevant for mitigating oxidative stress. Oxidative stress is a key factor in the development of chronic metabolic disorders, including diabetes mellitus. Antioxidants can help prevent oxidative damage to pancreatic β-cells. They may also improve insulin sensitivity by reducing reactive oxygen species. Therefore, the observed antioxidant activity supports the biological relevance of the extract. The DPPH assay provides a rapid and reliable evaluation of radical scavenging potential. The results confirm that the extract possesses substantial antioxidant capacity. This finding justifies further investigation of its phytochemical contributors.

**Table 2:** Antioxidant Activity and Phenolic–Flavonoid Content of *Rosa hybrida* Hort. Flower Ethanolic Extract*.*

**No**	**Analysis Parameters**	**Methods**	**Sample**	**Results (± SD)**	**Unit**	**Description**
1	Antioxidant Activity	DPPH	Ethanol extract of *Rosa hybrida* Hort.	9.775 ± 0.025	µg/mL (IC₅₀)	Very strong antioxidant
2	Antioxidant Activity	DPPH	Vitamin C (positive control)	3.909 ± 0.019	µg/mL (IC₅₀)	Very strong antioxidant
3	Total Phenolic Content (TPC)	Folin–Ciocalteu	Ethanol extract of *Rosa hybrida* Hort.	60.508	mg GAE/100 mg extract	Content high phenolic
4	Total Flavonoid Content (TFC)	AlCl₃ Colorimetric	Ethanol extract of *Rosa hybrida* Hort.	38.507	mg QE/100 mg extract	Content high flavonoid

The strong antioxidant activity of the extract is closely associated with its high total phenolic content (TPC) and total flavonoid content (TFC). Phenolic compounds are widely recognized as major contributors to antioxidant activity due to their redox properties. In this study, the extract exhibited a TPC of 60.508 mg GAE per 100 mg of extract, indicating a very high concentration of phenolic constituents. When converted to a comparable basis, this value corresponds to approximately 600 mg GAE per 100 g of extract. This phenolic level is higher than that reported for many commonly studied plant materials. Phenolic compounds exert antioxidant effects primarily through hydrogen atom transfer and electron transfer mechanisms. These mechanisms enable the neutralization of free radicals and termination of oxidative chain reactions. The Folin–Ciocalteu assay, although not entirely specific to phenolics, reflects the total reducing capacity of the extract. High reducing capacity generally correlates with strong antioxidant potential. In addition to phenolics, the extract demonstrated a high total flavonoid content of 38.507 mg QE per 100 mg of extract. This value indicates that flavonoids constitute a substantial portion of the total polyphenolic fraction. Flavonoids possess multiple hydroxyl groups that enhance their ability to donate hydrogen atoms. They can also stabilize free radicals through resonance structures. Certain flavonoids are capable of chelating pro-oxidant metal ions, further enhancing antioxidant protection. The combined presence of high TPC and TFC explains the low DPPH IC₅₀ value observed. Previous studies have demonstrated negative correlations between phenolic or flavonoid content and DPPH IC₅₀ values. These relationships indicate that higher polyphenol levels lead to stronger antioxidant activity. The results of this study are consistent with such findings. The phenolic and flavonoid richness of the extract therefore plays a central role in its antioxidant performance.

### 3.5 Alpha-glucosidase inhibitory activity dan alpha-amylase inhibitory activity

The *in vitro* α-glucosidase inhibitory activity was evaluated by assessing the ability of the red rose flower extract to suppress enzymatic hydrolysis of the substrate by α-glucosidase. The results presented in Table 3 demonstrate that the extract inhibited α-glucosidase activity in a clear dose-dependent manner. Increasing extract concentrations resulted in progressively higher inhibition percentages, indicating effective interactions between extract constituents and the enzyme. At the highest tested concentration of 100 ppm, α-glucosidase inhibition reached approximately 95.3%. In contrast, at the lowest concentration of 1.56 ppm, inhibition was only around 23.6%. The calculated IC₅₀ value of the extract was 4.45 ppm. This value indicates that a relatively low concentration of the extract is sufficient to inhibit 50% of α-glucosidase activity. This finding, together with the increase in inhibition from 23.58% to 95.25% across the tested concentration range, confirms a consistent concentration-dependent inhibitory response. Acarbose, used as the positive control, exhibited a markedly lower IC₅₀ value of approximately 0.01 ppm. This result reflects the exceptionally high potency of acarbose as a pharmaceutical α-glucosidase inhibitor. Compared with acarbose, the inhibitory potency of the red rose extract is lower. Approximately 445-fold higher extract concentration was required to achieve 50% enzyme inhibition. Despite this difference, an IC₅₀ value of 4.45 ppm is considered very strong for a crude plant extract. Many plant extracts reported in the literature show α-glucosidase IC₅₀ values in the tens to hundreds of micrograms per milliliter. Therefore, the inhibitory potency observed for the red rose extract can be regarded as relatively high. The strong α-glucosidase inhibition is consistent with the phytochemical profile of the extract. This profile includes abundant anthocyanins and flavonoids that are known to interfere with carbohydrate-digesting enzymes.

**Table 3:** *In Vitro* α-Glucosidase and α-Amylase Inhibitory Activities of *Rosa hybrida* Hort. Flower Extract.

**No**	**Target Enzyme**	**Sample**	**Concentration Range (ppm)**	**Percentage Inhibition (%)**	**IC₅₀ (ppm)**	**Activity Category**
1	α-Glucosidase	Extract of *Rosa hybrida* Hort.	1.56 – 100	23.58 – 95.25	4.45	Very strong
2	α-Glucosidase	Acarbose (positive control)	0.0008 – 0.1	1.65 – 87.42	0.01	Very strong
3	α-Amylase	Extract of *Rosa hybrida* Hort.	5,000 – 35000	16.85 – 87.81	17,149.28	Medium-Weak
4	α-Amylase	Acarbose (positive control)	625 – 20,000	19.63 – 66.10	8644.76	Medium

In addition to α-glucosidase inhibition, the red rose flower extract was also evaluated for its activity against α-amylase, an enzyme responsible for the initial hydrolysis of dietary starch. The extract exhibited a dose-dependent inhibitory pattern toward α-amylase. However, the inhibitory potency against α-amylase was markedly lower than that observed against α-glucosidase. At a concentration of 5,000 ppm, the extract inhibited only about 16–17% of α-amylase activity. The inhibitory activity progressively increased with increasing extract concentration. At the highest tested concentration of 35000 ppm, α-amylase inhibition reached around 88%. The calculated IC₅₀ value for α-amylase inhibition by the extract was 17,149.28 ppm. This value indicates that relatively high concentrations are required to inhibit 50% of α-amylase activity. Under the same experimental conditions, acarbose showed an IC₅₀ value of approximately 8644.76 ppm. Thus, acarbose was about twice as potent as the red rose extract in inhibiting α-amylase. This difference is consistent with the known pharmacological profile of acarbose, which exhibits stronger activity toward α-glucosidase than α-amylase. A similar selectivity pattern was observed for the red rose extract. The extract inhibited α-glucosidase at much lower concentrations than those required for α-amylase inhibition. This selectivity may be attributed to the dominance of anthocyanins and flavonoids rather than high-molecular-weight tannins. Anthocyanin-rich extracts are widely reported to preferentially inhibit α-glucosidase. Partial inhibition of α-amylase is still beneficial for regulating carbohydrate digestion. By suppressing starch hydrolysis and disaccharide breakdown at different stages, dual enzyme inhibition may reduce postprandial glucose spikes. Therefore, the combined inhibitory profile supports the potential of red rose flower extract as a natural antidiabetic supplement.

### 3.6 LC-MS/MS profiling

**Fig. 1:**
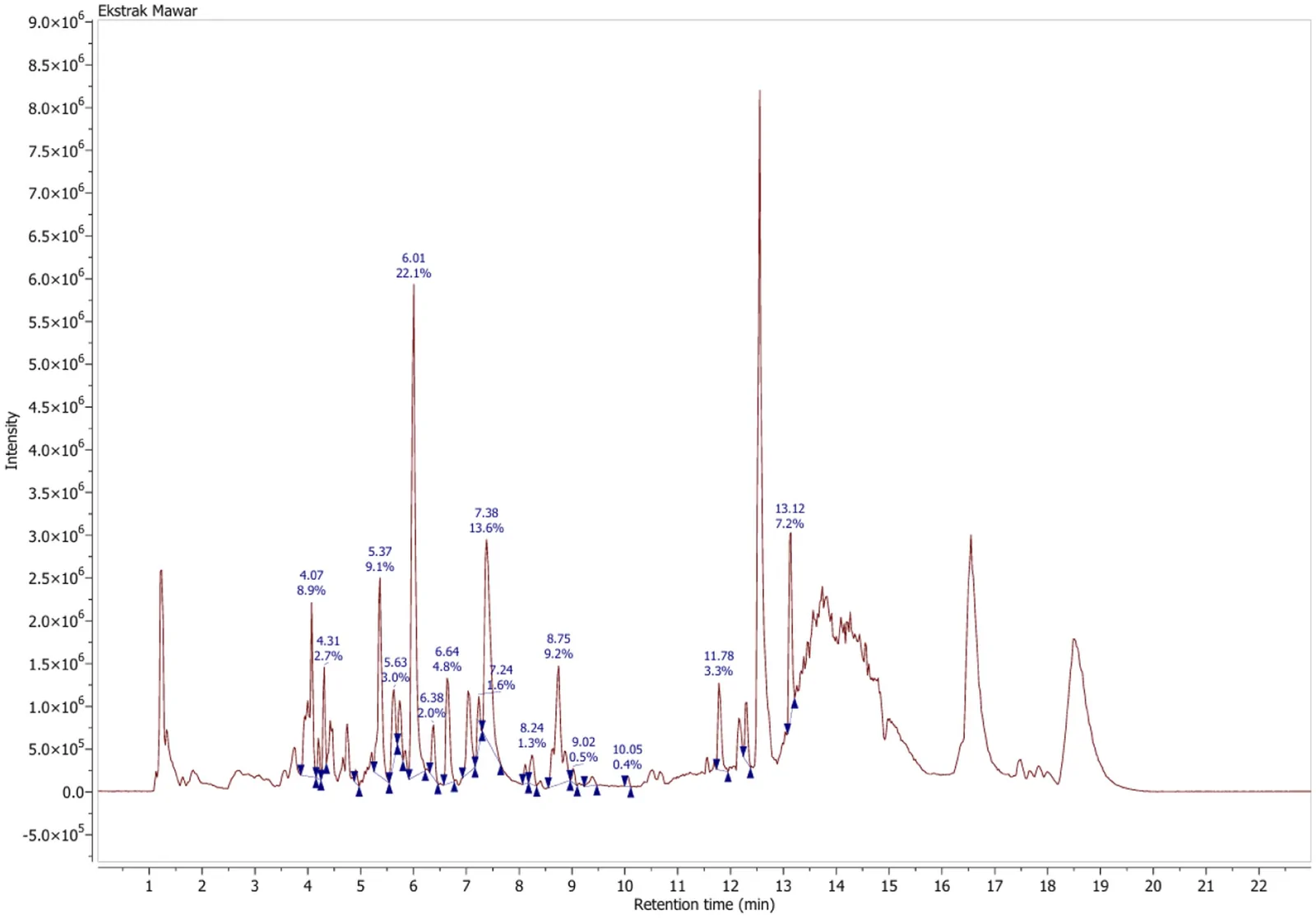
LC–MS/MS Chromatographic Profile of *Rosa hybrida* Hort. Flower Ethanolic Extract.

LC–MS/MS analysis of the red rose flower extract (*Rosa hybrida* Hort.) revealed a complex metabolite profile with at least 22 detected compound peaks across a wide range of retention times and mass-to-charge ratios. The chromatographic separation shown in Fig. 1 reflects the presence of multiple secondary metabolites with varying polarity and molecular structures. Such diversity is characteristic of plant-derived extracts rich in specialized metabolites. The detected compounds were classified into flavonoids, anthocyanins, phenolamides, terpenoids, and several minor constituents. This chemical diversity provides a molecular explanation for the biological activities observed in the extract. Phenolic compounds were predominant, supporting the strong antioxidant activity demonstrated in the DPPH assay. Phenolics are known to neutralize reactive oxygen species through hydrogen atom and electron donation mechanisms. Their abundance also aligns with the potent inhibition of α-glucosidase reported for this extract. Metabolite profiling therefore connects chemical composition with biological function. Polyhydroxylated structures are particularly effective as radical scavengers. These structures are also capable of forming hydrogen bonds with enzyme active sites. The LC–MS/MS data confirm that the extract contains multiple bioactive constituents rather than a single dominant compound. Such multiplicity allows different molecular targets to be modulated simultaneously. This property is advantageous in the context of metabolic disorders. The extract composition supports multitarget biological activity. The LC–MS/MS profile is consistent with the phytochemical screening results. These findings strengthen the interpretation of the extract’s pharmacological relevance. The metabolite diversity underscores the importance of comprehensive chemical characterization.

Flavonoids represented the most prominent group of compounds detected in the extract, appearing as both aglycones and glycosides, as summarized in Table 4. Flavonol aglycones such as quercetin and kaempferol were detected in relatively high proportions. These compounds are widely recognized as potent antioxidants due to their polyphenolic structures. Multiple hydroxyl groups on their aromatic rings enable efficient hydrogen and electron donation. The high relative abundance of kaempferol and quercetin is consistent with the very strong DPPH radical scavenging activity observed. In addition to antioxidant effects, these flavonols have been extensively reported to inhibit α-glucosidase. They can interact with catalytic residues through hydrogen bonding and hydrophobic interactions. Such interactions interfere with substrate binding and enzymatic catalysis. Several flavonoid glycosides were also identified, including hyperoside, juglanin, and multiflorins A and B. These glycosylated flavonoids exhibit higher polarity and are readily extracted by ethanol. Although glycosylation may reduce direct antioxidant potency, these compounds act as biologically relevant precursors. Enzymatic hydrolysis *in vivo* can convert glycosides into active aglycones. Some flavonoid glycosides have also been reported to directly inhibit carbohydrate-digesting enzymes. The coexistence of aglycones and glycosides suggests both immediate and sustained biological effects. This compositional feature enhances the functional potential of the extract. Flavonoids therefore represent a major molecular contributor to antioxidant and antidiabetic activities. Their dominance explains the strong enzyme inhibition observed experimentally.

**Table 4:** LC–MS/MS–Based Identification of Major Metabolites in *Rosa hybrida* Hort. Flower Ethanolic Extract.

**No**	**RT (min)**	**Parrent (*m/z*)**	**Molecular** **Weight**	**Molecular** **Formula**	**Compound Name**	**Compound Composition (%)**
1	4.07	662.1920		C_27_H_35_NO_18_		8.9
2	4.20	500.1385	499.30	C_26_H_45_NO_6_S	Taurodeoxycholate	1.0
3	4.31	420.1876	419.10	C_20_H_19_O_10_^+^	Cyanidin-3-O-alpha-arabinopyranoside	2.7
4	4.92	465.1017	464.38	C_21_H_20_O_12_	Hyperoside	4.4
5	5.37	303.0510	302.24	C_15_H_10_O_7_	Quercetin	9.1
6	5.63	595.1715	594.52	C_27_H_30_O_15_	Multiflorin B	3.0
7	5.74	419.0996	418.35	C_20_H_18_O_10_	Juglanin	1.8
8	6.01	287.0574	286.24	C_15_H_10_O_6_	Kaempherol	22.1
9	6.38	191.1423	190.28	C_13_H_18_O	Damascenone	2
10	6.64	637.1756	636.56	C_29_H_32_O_16_	Multiflorin A	4.8
11	7.03	600.2701		C_31_H_30_N_13_O		4.2
12	7.24	309.0868	196.24	C_11_H_16_O_3_		1.6
13	7.38	584.2766	583.68	C_34_H_37_N_3_O_6_	Tricoumaroyl Spermidine	13.6
14	8.12	221.1902	220.35	C_15_H_24_O	Dehydroxy-isocalamendiol	0.6
15	8.24	275.2019	274.40	C_18_H_26_O_2_	13-Methoxy-14-hydroxypodocarpa-8,11,13-triene	1.3
16	8.75	584.2752		C_34_H_37_N_3_O_6_		9.2
17	9.02	508.3137	507.26	C_23_H_45_N_3_O_9_	Cytochalasin	0.5
18	9.38	353.2303	352.47	C_20_H_32_O_5_	1-(3,4-Dimethoxyphenyl)-5-acetoxy-3-decanol	0.6
19	10.05	343.296	342.29	C_19_H_38_N_2_O_3_	Cocamidopropyl betaine	0.4
20	11.78	518.3221		C_23_H_43_N_5_O_8_		3.3
21	12.3	520.3411		C_26_H_50_NO_9_		1.9
22	13.14	496.3388		C_21_H_45_N_5_O_8_		7.2

Anthocyanins were detected in the extract, particularly cyanidin-3-O-α-arabinopyranoside, which is responsible for the characteristic red coloration of rose petals. Anthocyanins are flavonoid pigments known for strong antioxidant and anti-inflammatory properties. These compounds are capable of scavenging free radicals and modulating oxidative stress pathways. Anthocyanins have also been reported to exhibit high affinity toward α-glucosidase. Their presence may therefore contribute to the strong enzyme inhibitory activity observed. A notable finding was the detection of phenolamides, especially tricoumaroyl spermidine, appearing as multiple peaks with identical molecular formulas but different retention times. This pattern indicates the presence of structural isomers. Phenolamides are conjugates of hydroxycinnamic acids and polyamines. They function as plant defense metabolites and possess significant antioxidant activity. Their conjugated aromatic systems facilitate radical stabilization. Phenolamides have also been reported to interact with enzymes and proteins. The relatively high abundance of tricoumaroyl spermidine suggests an important contribution to biological activity. Several terpenoids were detected in smaller amounts, including sesquiterpenes and diterpenes. These compounds are known for anti-inflammatory and antimicrobial properties. The detection of β-damascenone supports the presence of aroma-related constituents. Some minor detected compounds likely represent analytical artifacts or contaminants. Careful interpretation ensures biological relevance is attributed only to genuine plant metabolites. The LC–MS/MS data collectively support the link between chemical composition and observed antioxidant and antidiabetic activities.

### 3.7 Preparation of test ligands

The data presented in Table 5 describe the chemical structures and SMILES representations of selected bioactive compounds identified from the *Rosa hybrida* Hort. flower extract and used for subsequent *in silico* analysis. These compounds were selected based on their relative abundance, structural diversity, and reported biological relevance. The selected molecules include flavonoid aglycones, flavonoid glycosides, phenolic derivatives, and a representative terpenoid. Flavonoid aglycones such as quercetin and kaempferol exhibit a classical flavonol backbone characterized by multiple aromatic hydroxyl groups. These hydroxyl substituents are critical structural features that facilitate hydrogen bonding and electron donation. Such features are closely associated with antioxidant activity and enzyme inhibition. The planar aromatic rings of flavonols also allow π–π stacking interactions with aromatic residues in enzyme active sites. These interactions are particularly relevant for α-glucosidase inhibition. The SMILES representations confirm the presence of multiple hydrogen bond donors and acceptors. This structural richness supports strong ligand–protein interactions during molecular docking simulations. Flavonoid glycosides such as multiflorin A, multiflorin B, and juglanin contain additional sugar moieties attached to the flavonoid core. Glycosylation increases molecular polarity and affects steric orientation within enzyme binding pockets. Although glycosides are bulkier than aglycones, they still retain several hydroxyl groups capable of forming hydrogen bonds. The presence of both aglycone and glycoside forms suggests variability in binding modes. This variability is advantageous for exploring different interaction patterns with target enzymes. The inclusion of these compounds provides structural representation of major phenolic classes present in the extract. Their selection is therefore chemically and biologically justified.

**Table 5:** Chemical Structures and SMILES Representation of Selected Bioactive Compounds from *Rosa hybrida* Hort. Flower Extract.

**Code**	**Compound Name**	**2D Structure**	**SMILES**
S01	Taurodeoxycholate		CC(CCC(=O)NCCS(=O)(=O)O)C1CCC2C1(C(CC3C2CCC4C3(CCC(C4)O)C)O)C
S02	Cyanidin-3-O-alpha-arabinopyranoside		C1C(C(C(C(O1)OC2 =CC3 =C(C=C(C=C3[O+]=C2C4 =CC(=C(C=C4)O)O)O)O)O)O)O
S03	Hyperoside		C1 =CC(=C(C=C1C2 =C(C(=O)C3 =C(C=C(C=C3O2)O)O)OC4C(C(C(C(O4)CO)O)O)O)O)O
S04	Quercetin		C1 =CC(=C(C=C1C2 =C(C(=O)C3 =C(C=C(C=C3O2)O)O)O)O)O
S05	Multiflorin B		CC1C(C(C(C(O1)OC2 =C(OC3 =CC(=CC(=C3C2 =O)O)O)C4 =CC=C(C=C4)O)O)O)OC5C(C(C(C(O5)CO)O)O)O
S06	Juglanin		C1 =CC(=CC=C1C2 =C(C(=O)C3 =C(C=C(C=C3O2)O)O)OC4C(C(C(O4)CO)O)O)O
S07	Kaempherol		C1 =CC(=CC=C1C2 =C(C(=O)C3 =C(C=C(C=C3O2)O)O)O)O
S08	Damascenone		CC=CC(=O)C1 =C(C=CCC1(C)C)C
S09	Multiflorin A		CC1C(C(C(C(O1)OC2 =C(OC3 =CC(=CC(=C3C2 =O)O)O)C4 =CC=C(C=C4)O)O)O)OC5C(C(C(C(O5)COC(=O)C)O)O)O
S10	Tricoumaroyl Spermidine		C1 =CC(=CC=C1C=CC(=O)NCCCCN(CCCNC(=O)C=CC2 =CC=C(C=C2)O)C(=O)C=CC3 =CC=C(C=C3)O)O
S11	Dehydroxy-isocalamendiol		CC1CC=C(C2(C1CCC(=C)C2)O)C(C)C
S12	13-Methoxy-14-hydroxypodocarpa-8,11,13-triene		COC1 =CC=C([C@](CCCC2(C)C)(C)[C@@]2([H])CC3)C3 =C1O
S13	Cytochalasin		CC1CCCC(C=CC(=O)OC23C(C=CC1)C4C(O4)(C(C2C(NC3 =O)CC5 =CC=CC=C5)C)C)O
S14	1-(3,4-Dimethoxyphenyl)-5-acetoxy-3-decanol		CCCCCC(OC(C)=O)CC(O)CCC1 =CC=C(OC)C(OC)=C1
S15	Cocamidopropyl betaine		CCCCCCCCCCCC(=O)NCCC[N + ](C)(C)CC(=O)[O-]

In addition to flavonoids, the dataset includes damascenone, a norisoprenoid compound with a relatively simple and lipophilic structure. Unlike polyphenols, damascenone lacks multiple hydroxyl groups and is less likely to form extensive hydrogen bonding interactions. However, its hydrophobic framework may contribute to van der Waals and hydrophobic contacts with protein residues. Such interactions can still influence ligand binding stability. The presence of damascenone highlights the chemical diversity of metabolites present in the extract. This diversity allows evaluation of how different chemical scaffolds interact with target enzymes. The SMILES notation further illustrates differences in molecular size, polarity, and functional group distribution among the selected compounds. Quercetin and kaempferol exhibit compact but highly functionalized structures. In contrast, multiflorin derivatives show increased molecular complexity due to glycosidic substitutions. These differences influence molecular flexibility and docking orientation. Structural flexibility is an important factor in molecular recognition. Compounds with multiple rotatable bonds may adopt favorable conformations within enzyme active sites. The combination of rigid aromatic systems and flexible sugar chains can enhance binding adaptability. The selected compounds therefore cover a broad chemical space relevant to enzyme inhibition. This structural variation supports comparative interpretation of docking results. It also aids in rationalizing differences in predicted binding energies and interaction profiles. The chemical information provided forms a solid molecular basis for subsequent *in silico* evaluation.

### 3.8 Molecular docking validation

Molecular docking was conducted using AutoDock version 4.2.3 to investigate ligand–protein interactions involving α-glucosidase and α-amylase. The crystal structures of both target proteins were retrieved from the RCSB Protein Data Bank. These protein structures initially contained crystallographic water molecules originating from X-ray diffraction analysis. All water molecules were removed prior to docking to prevent steric interference with ligand binding. Removal of water molecules ensures that predicted interactions occur solely between ligands and protein residues. This step is commonly applied in molecular docking studies to improve interaction accuracy. The protein structures determined by X-ray crystallography lacked hydrogen atoms in their original files. Polar hydrogen atoms were therefore added to the receptors before docking. Polar hydrogens are essential for accurately modeling hydrogen bond interactions. Hydrogen bonding plays a crucial role in ligand recognition and binding stability. Only polar hydrogens were included in the receptor preparation. Nonpolar hydrogens were excluded to reduce computational complexity. The addition of polar hydrogens improves electrostatic representation of the binding site. Proper receptor preparation is critical for reliable docking simulations. Inaccurate preparation may lead to erroneous binding predictions. The prepared protein structures were subsequently converted into AutoDock-compatible file formats. This preparation protocol follows standard molecular docking guidelines. Consistent receptor preparation enhances reproducibility across simulations. The applied procedure ensured methodological consistency for both target proteins. These steps provided a robust foundation for docking validation.

Docking validation was performed using a redocking approach, in which native ligands were docked back into their respective protein binding sites. Redocking is a widely accepted method for evaluating docking accuracy. The resulting ligand conformations were compared with their crystallographic binding poses. The degree of similarity between these conformations was quantified using root mean square deviation (RMSD). RMSD is a standard metric for assessing docking reliability. Lower RMSD values indicate closer reproduction of experimental ligand positions. In this study, RMSD values obtained for both target proteins were within the accepted threshold. As summarized in Table 6, all RMSD values were below 2.0 Å. This threshold is widely regarded as the criterion for successful docking validation. The low RMSD values indicate minimal deviation between docked and crystallographic ligand poses. These results confirm that the docking protocol accurately reproduced native binding modes. Correct reproduction of native ligand positions validates grid center selection. It also confirms appropriate grid box dimensions. The validation results demonstrate that the docking parameters were correctly defined. Reliable validation is essential before docking test ligands. Without validation, docking predictions may lack credibility. The successful redocking results support the accuracy of the docking workflow. This validation step confirms methodological soundness. The protocol can therefore be used confidently for subsequent simulations.

**Table 6:** Molecular Docking Validation Parameters for α-Glucosidase and α-Amylase Targets.

**PDB Code**	**Grid Center**			**Grid Box**			**RMSD**
	**x**	**y**	**z**	**x**	**y**	**z**	
5NN8	-8.284	-33.342	90.637	52	76	52	1.479 Å
1B2Y	17.388	8.830	49.691	54	64	50	1.651 Å

Docking simulations were performed using the Lamarckian Genetic Algorithm, which integrates global search and local optimization strategies. This algorithm is widely regarded as one of the most accurate docking methods. It efficiently explores ligand conformational space within the binding site. In this study, the number of genetic algorithm runs was set to 100. A high number of runs increases the likelihood of identifying the global minimum energy conformation. Each run generates a distinct ligand–protein interaction pose. These poses are evaluated and ranked based on predicted binding energy. Ranking allows identification of the most favorable binding orientation. Grid box parameters were defined based on validated active site regions. The grid boxes encompassed the entire catalytic pockets of the target proteins. Proper grid definition is critical for accurate docking. An undersized grid may restrict ligand movement. An oversized grid may reduce computational efficiency. The selected grid dimensions balanced flexibility and precision. This configuration allowed ligands to explore all relevant binding orientations. Adequate sampling improves prediction reliability. Grid box placement was centered on known active site residues. This ensured biologically relevant interaction sampling. The docking setup allowed sufficient ligand flexibility. These optimized parameters enhanced docking robustness.

**Fig. 2:**
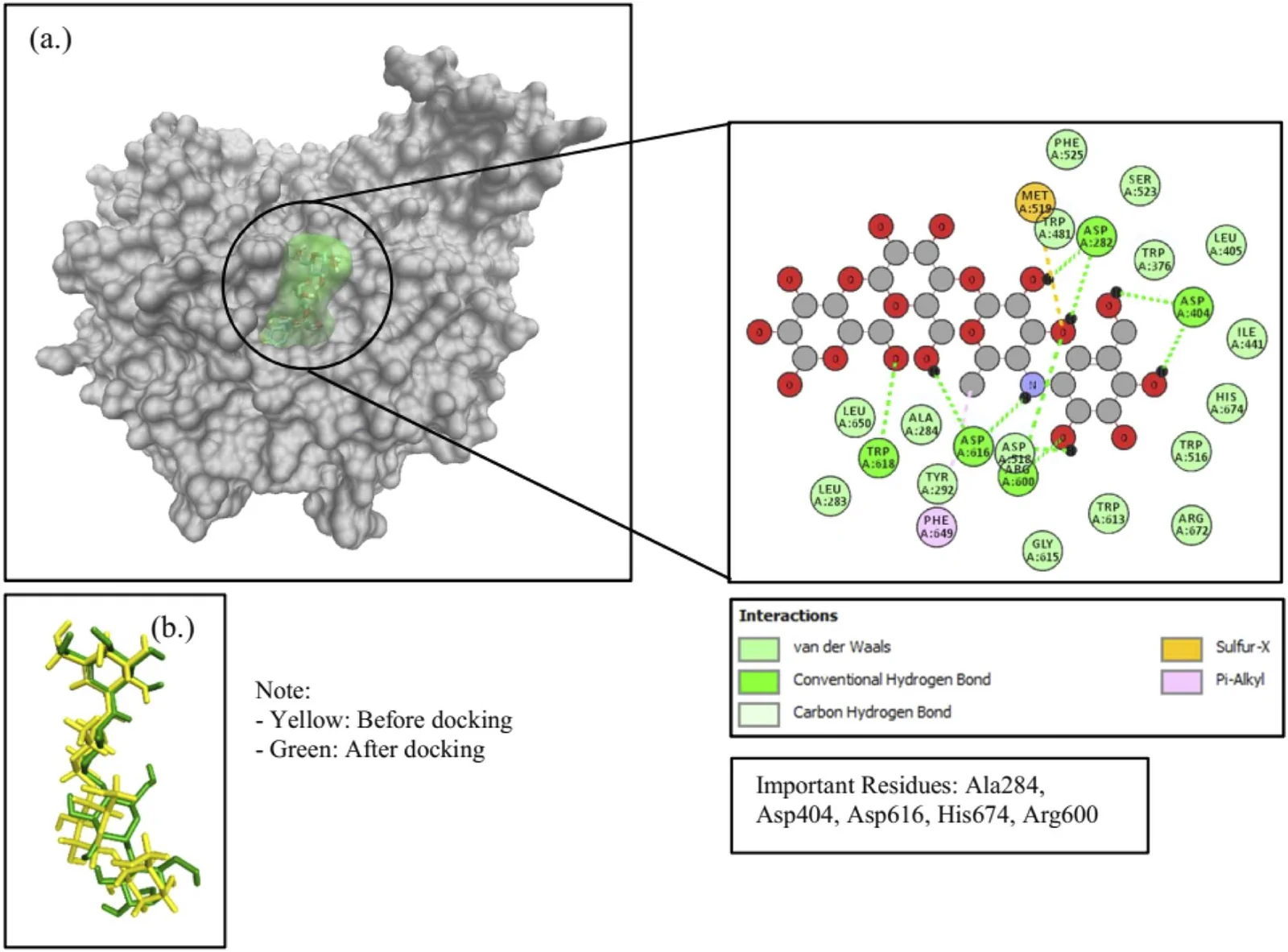
Molecular Docking Validation of the Native Ligand on the α-Glucosidase Protein: (a) Binding Site Visualization and (b) Overlay of the Native Ligand Before and After Docking.

**Fig. 3:**
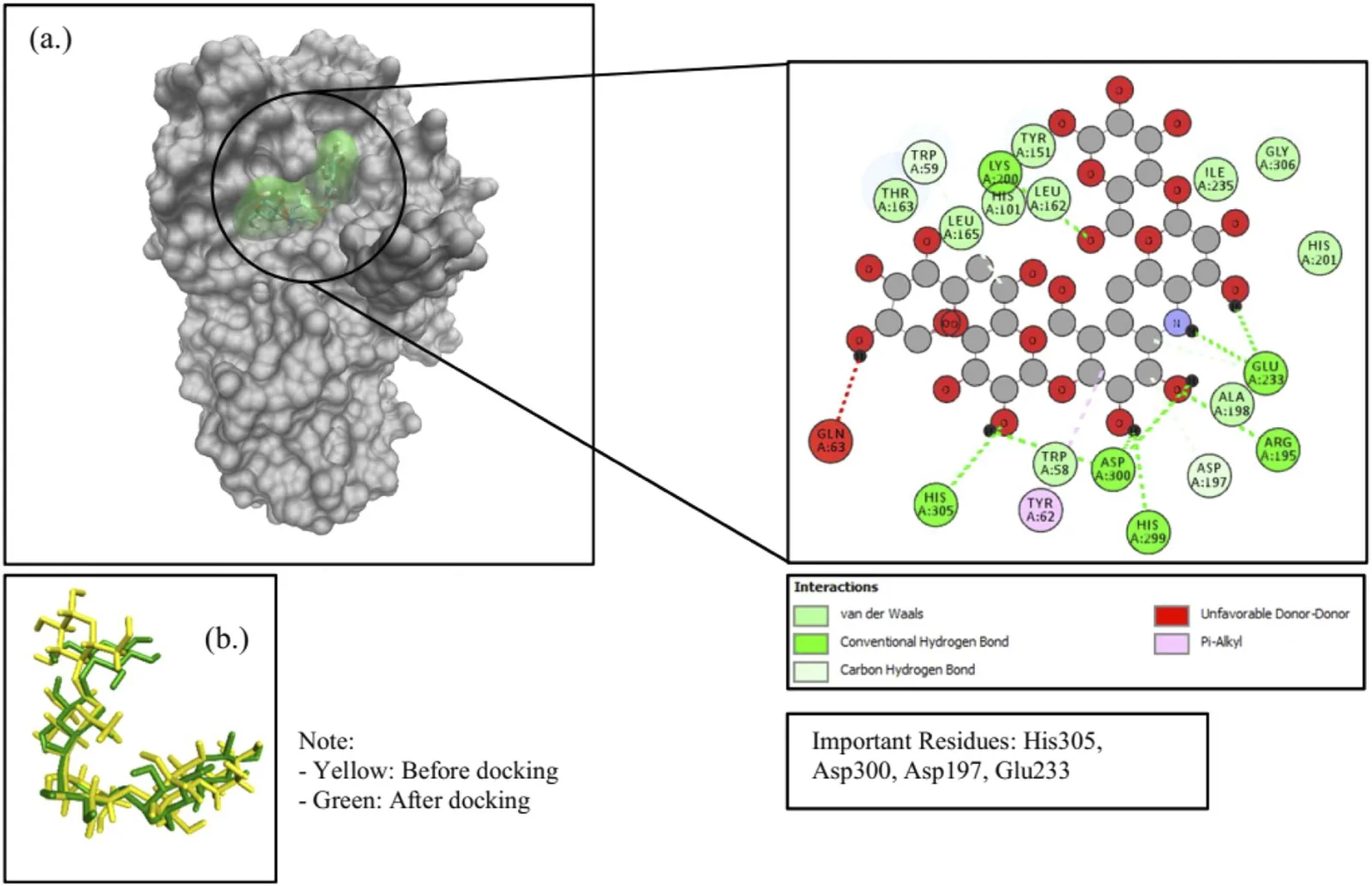
Molecular Docking Validation of the Native Ligand on the α-Amylase Protein: (a) Binding Site Visualization and (b) Overlay of the Native Ligand Before and After Docking.

Visualization of the redocking results further supported the reliability of the docking protocol, as shown in Fig. 2 and Fig. 3. The overlay of native ligands before and after docking demonstrated high spatial congruence. This overlap indicates that ligand orientation was preserved after docking. Minimal positional deviation was observed within the binding pockets. For α-glucosidase, the redocked ligand maintained interactions with key catalytic residues. These residues are known to be essential for enzymatic activity. For α-amylase, conserved interactions with important active site residues were also preserved. Hydrogen bonds and van der Waals interactions were consistently observed. These interactions are fundamental for stable ligand binding. Preservation of these contacts indicates biologically relevant docking poses. The ligands occupied the correct binding cavities. No significant displacement from the active site was detected. This confirms accurate grid placement. Visualization complements RMSD-based validation. Both qualitative and quantitative analyses support protocol accuracy. The combined results demonstrate methodological reliability. The validated docking protocol can be applied to test ligands. It is suitable for evaluating bioactive compounds from *Rosa hybrida*. The validation ensures meaningful interpretation of binding affinity data. This approach provides a solid basis for further *in silico* investigations.

### 3.9 Molecular docking analysis of selected ligands against α-glucosidase

The molecular docking results against α-glucosidase reveal clear differences in binding affinity and interaction patterns among the evaluated ligands, as summarized in Table 7. The native ligand (NL) exhibited the most favorable binding energy of −9.30 kcal/mol and the lowest inhibition constant of 152.47 nM, indicating very strong affinity toward the enzyme. This high affinity is supported by the formation of ten hydrogen bonds involving critical residues such as Asp616, Asp404, Asp282, Arg600, and Trp618. These residues are well recognized as key contributors to the catalytic function and substrate stabilization of α-glucosidase. In addition to hydrogen bonding, hydrophobic interactions with Met519 and Phe649 further stabilized the native ligand within the binding pocket. Among the tested compounds, ligand S01 demonstrated a binding energy of −7.00 kcal/mol with a Ki value of 7.39 µM, indicating strong inhibitory potential. Ligand S01 formed six hydrogen bonds, mainly with Asp616, Asp404, and Asp282, highlighting its ability to anchor effectively in the catalytic region. Ligand S06 also showed a favorable binding profile with a binding energy of −6.91 kcal/mol and a Ki of 8.60 µM. The interaction pattern of S06 involved six hydrogen bonds with Asp616, Asp518, Asp404, Asp282, and Ala284, closely resembling the interaction network of the native ligand. Ligands S02 and S05 displayed moderate binding energies and inhibition constants in the low micromolar range, reflecting meaningful but reduced inhibitory strength. These ligands maintained several hydrogen bonds with key acidic residues, although fewer than those observed for S01 and S06. Ligands S03, S04, and S07 exhibited weaker binding energies and higher Ki values, indicating diminished affinity toward α-glucosidase. Their interaction profiles showed fewer hydrogen bonds and less optimal positioning within the active site. The gradual decrease in binding affinity across the ligand series corresponds to reduced stabilization within the catalytic pocket.

**Table 7:** Molecular Docking Results of Selected Ligands Against α-Glucosidase.

**Ligand**	**Binding Energy (kcal/mol)**	**Inhibition Constant**	**Number of Hydrogen Bond (HB)**	**Number of Hydrophobic Interaction (HI)**
NL	-9.30 kcal/mol	152.47 nM	10 HB with Trp618, **Asp616**, **Arg600**, Asp518, **Asp404**, Asp282	2 HI with Met519, Phe649
S01	-7.00 kcal/mol	7.39 uM	6 HB with **Asp616**, Arg281, **Asp404**, Asp282	6 HI with Met519, Phe649, Trp376, Trp481
S02	-6.65 kcal/mol	13.40 uM	5 HB with **Asp616**, Arg281, **Asp404**, Trp481	6 HI with Met519, **Asp616**, Trp376, Leu650
S03	-5.83 kcal/mol	53.34 uM	6 HB with **Asp616**, **Arg600**, **Asp404**, Asp282	4 HI with **Asp616**, Trp481, Phe649
S04	-5.66 kcal/mol	71.35 uM	4 HB with **Asp404**, Asp282, Ser523	8 HI with Met519, **Asp616**, Trp481, Phe649, Asp282
S05	-6.56 kcal/mol	15.59 uM	7 HB with **Asp616**, Trp618, **Asp404**, Asp282, Asp518	4 HI with Leu283, Leu650, Ala284
S06	-6.91 kcal/mol	8.60 uM	6 HB with **Asp616**, Asp518, **Asp404**, Asp282, Ala284, Trp481	6 HI with Met519, **Asp616**, Trp376, Leu650
S07	-5.88 kcal/mol	49.10 uM	5 HB with **Asp616**, Arg281, Asn524, Trp481, Ala284	5 HI with Met519, **Asp616**, Trp481, **Asp282,** Ala555
S08	-6.11 kcal/mol	33.19 uM	0 HB	9 HI with Trp481, Phe649, Trp376, Leu405, **His674**
S09	-5.96 kcal/mol	42.52 uM	5 HB with **Asp616**, Asp518, **Asp404**, **Arg600**, Ala284	4 HI with Met519, **Asp616**, Trp481
S10	-6.20 kcal/mol	28.61 uM	2 HB with Thr286, Asp404	3 HI with Asp518, Phe649, **Ala284**
S11	-6.47 kcal/mol	18.18 uM	1 HB with Asp518	10 HI with Trp481, Phe649, Trp376, Trp516, Trp613, **His674**
S12	-6.03 kcal/mol	37.88 uM	1 HB with Asp282	12 HI with Trp481, Phe649, Trp376, Trp516, Trp613, **His674,** Ile441, **Asp616,** Met519
S13	-3.75 kcal/mol	1.77 mM	2 HB with Asp518, **Arg600**	4 HI with Phe649, Leu650, Phe525, Asp282
S14	-5.42 kcal/mol	105.88 uM	2 HB with Asp282, Arg281	5 HI with Met519, Ile441, Trp481, **Asp282,** Leu405
S15	-5.42 kcal/mol	106.17 uM	1 HB with Asp282	5 HI with Arg281, Ile441, Trp481, **Asp282,** Leu405

An examination of the interaction profiles indicates that hydrogen bonding is a dominant determinant of ligand potency against α-glucosidase. Ligands forming multiple hydrogen bonds with Asp616, Asp404, and Asp282 consistently showed lower inhibition constants and more favorable binding energies. These acidic residues are essential for enzymatic catalysis, and their engagement by inhibitors can effectively disrupt substrate processing. In contrast, ligands relying primarily on hydrophobic interactions tended to exhibit weaker inhibitory performance. For example, ligand S08 showed several hydrophobic contacts but formed no hydrogen bonds, which was reflected in its higher Ki value. This observation suggests that hydrophobic interactions alone are insufficient to ensure strong enzyme inhibition. Ligands such as S11 and S12 exhibited extensive hydrophobic interactions with aromatic residues including Trp481, Trp376, and Phe649. Despite these contacts, their inhibition constants remained higher than those of ligands enriched in hydrogen bonding. The balance between hydrogen bonding and hydrophobic stabilization appears critical for optimal binding. Ligands with limited functional groups capable of hydrogen bond donation or acceptance showed reduced affinity and stability within the active site. Structural differences among the ligands, including substituent type and molecular flexibility, contributed to variability in binding behavior. Several test ligands were still able to reproduce key interaction motifs observed for the native ligand, albeit with lower strength. This indicates that these compounds can access the catalytic pocket and adopt plausible inhibitory conformations. The recurrent involvement of Asp616 and Asp404 across multiple ligands underscores their importance as anchoring residues. The docking results therefore provide a consistent molecular explanation for the differences in predicted inhibitory activity. Ligands S01 and S06 emerge as the most promising candidates based on their binding energies, inhibition constants, and interaction networks. Their ability to form stable hydrogen-bonded complexes supports their potential relevance as α-glucosidase inhibitors.

### 3.10 Molecular docking analysis of selected ligands against α-amylase

Molecular docking analysis was conducted to evaluate the binding affinity and interaction characteristics of selected ligands against α-amylase, an enzyme responsible for the initial hydrolysis of dietary starch. The docking results summarized in Table 8 show distinct variations in binding energy, inhibition constant (Ki), and interaction profiles among the tested ligands. The native ligand (NL) demonstrated a favorable binding energy of −9.60 kcal/mol with a Ki value of 91.16 nM, indicating strong inhibitory potential toward α-amylase. This affinity was supported by the formation of seven hydrogen bonds involving key catalytic residues such as Glu233, Asp300, Thr163, and His305. These residues are widely recognized for their roles in substrate binding and catalytic activity. Additional hydrophobic interactions with Tyr62 and Leu165 contributed to stabilization of the ligand within the active site. Among the tested compounds, ligand S13 exhibited the most favorable binding energy of −9.82 kcal/mol and the lowest Ki value of 63.70 nM. Despite forming only two hydrogen bonds, the low Ki value of S13 suggests strong stabilization through optimal orientation and complementary interactions. Ligand S01 also showed strong affinity with a binding energy of −9.19 kcal/mol and a Ki of 182.81 nM. The interaction network of S01 consisted of six hydrogen bonds and five hydrophobic interactions, indicating balanced binding stabilization. Ligands S02 and S10 displayed binding energies of −8.62 and −8.60 kcal/mol, respectively, with Ki values in the submicromolar range. Their interaction profiles included extensive hydrogen bonding with residues such as Glu233, Asp197, and Asp300. These residues are located within the catalytic pocket and are critical for enzymatic function. Several tested ligands achieved binding affinities comparable to the native ligand. The presence of multiple acidic residues in ligand–enzyme interactions highlights the importance of electrostatic contributions to α-amylase inhibition. The variation in binding energies reflects differences in molecular size, polarity, and functional group distribution. These results demonstrate that certain test ligands are capable of forming stable complexes within the α-amylase active site.

**Table 8:** Molecular Docking Results of Selected Ligands Against α-amylase.

**Ligand**	**Binding Energy (kcal/mol)**	**Inhibition Constant**	**Number of Hydrogen Bond (HB)**	**Number of Hydrophobic Interaction (HI)**
NL	-9.60 kcal/mol	91.16 nM	7 HB with: **Glu233**, Arg195, **Asp300**, Gln63, Thr163, **His305**	2 HI with: Tyr62, Leu165
S01	-9.19 kcal/mol	182.81 nM	6 HB with: **Glu233**, Ile234, Lys200, His201	5 HI with: Trp59, Leu165, **His305**
S02	-8.62 kcal/mol	480.79 nM	8 HB with: **Glu233**, **Asp197**, **Asp300**, Ile235, Lys200	7 HI with: Ile235, **Glu233,** Ala198, His201, Leu162, Lys200
S03	-8.10 kcal/mol	1.15 uM	8 HB with: **Glu233**, **Asp197**, His299, Ile235, Gly305	7 HI with: Ile235, **Glu233,** Ala198, His201, Leu162, Lys200
S04	-7.00 kcal/mol	7.39 uM	4 HB with: **Glu233**, **Asp197**, His299, Ile235	10 HI with: Ile235**,** Ala198, His201, Leu162, Lys200, **Asp300**, Tyr62
S05	-8.10 kcal/mol	1.16 uM	5 HB with: **Glu233**, Tyr62, Thr163, Ile235	7 HI with: Ile235, **Glu233,** Ala198, His201, Lys200
S06	-7.97 kcal/mol	1.45 uM	8 HB with: **Glu233**, **Asp197**, Thr163, Ile235, **Asp300**	6 HI with: Ile235, **Glu233,** Ala198, His201, Lys200, Leu162
S07	-6.50 kcal/mol	17.25 uM	7 HB with: **Glu233**, Arg195, **Asp300**, Gln63, Thr163, **His305**	5 HI with: Ile235, Ala198, His201, Lys200, Leu162
S08	-6.05 kcal/mol	36.90 uM	1 HB with: Gln63	8 HI with: Leu165, Trp58, Tyr62, His305, Trp59
S09	-8.22 kcal/mol	938.45 nM	7 HB with: **Glu233**, Arg195, **Asp300**, Gln63, Thr163, **His305**	11 HI with: Ile235**,** Ala198, His201, Leu162, Lys200, **Asp300**, Tyr62
S10	-8.60 kcal/mol	498.80 nM	5 HB with: **Glu233**, Arg195, **Asp300,** Lys200	5 HI with: Ile235, Ala198, His201, Lys200, Trp59
S11	-6.40 kcal/mol	20.42 uM	2 HB with: Gln63, Trp59	6 HI with: Trp58, Trp59, His101, Leu165
S12	-7.39 kcal/mol	3.85 uM	1 HB with: Tyr62	6 HI with: Trp58, Tyr62, His299, Leu162, Ala198
S13	-9.82 kcal/mol	63.70 nM	2 HB with: **His305, Asp197**	1 HI with: Leu162
S14	-6.36 kcal/mol	21.87 uM	2 HB with: **His305, Asp197**	4 HI with: Ile235, Ala198, Leu162, Trp59
S15	-5.43 kcal/mol	104.20 uM	1 HB with: **Glu233**	3 HI with: Lys200, **His305**, Trp58

A closer analysis of the interaction patterns indicates that hydrogen bond formation is a primary determinant of inhibitory potency against α-amylase. Ligands forming multiple hydrogen bonds with catalytic residues such as Glu233, Asp197, Asp300, and His305 generally exhibited lower Ki values and stronger binding energies. Ligands S02, S03, and S06 each formed eight hydrogen bonds and showed binding energies between −7.97 and −8.62 kcal/mol, indicating stable ligand–enzyme complexes. In contrast, ligands that relied predominantly on hydrophobic interactions tended to show reduced inhibitory potency. Ligand S08 formed only one hydrogen bond and displayed a Ki value of 36.90 µM, reflecting weak inhibition. This suggests that hydrophobic contacts alone are insufficient for effective α-amylase inhibition. Ligands S04 and S07 exhibited moderate binding energies and Ki values in the micromolar range, corresponding to fewer stabilizing interactions. Ligand S12 showed a binding energy of −7.39 kcal/mol and formed only one hydrogen bond, resulting in a Ki value of 3.85 µM. Ligand S15 exhibited the weakest binding profile with the highest Ki value of 104.20 µM. Differences in interaction patterns are influenced by ligand flexibility, steric compatibility, and functional group orientation. Several ligands reproduced key interaction motifs observed for the native ligand but with reduced strength. Recurrent interactions with Glu233 and Asp300 emphasize their importance as anchoring residues. The correlation between binding energy, hydrogen bonding, and Ki values is evident across the ligand series. Ligands S13, S01, S02, and S10 demonstrate particularly favorable interaction characteristics. These compounds show potential relevance for further investigation as α-amylase inhibitors.

## 4 Discussions

The extraction of *Rosa hybrida* Hort. flowers using ethanolic maceration proved effective in recovering bioactive secondary metabolites relevant to antidiabetic activity. Ethanol was selected as the solvent due to its ability to dissolve a broad range of polar and semi-polar compounds, including flavonoids, anthocyanins, tannins, and phenolamides. The mild extraction conditions minimized thermal degradation of thermolabile constituents, particularly polyphenols. This was reflected in the high total phenolic and flavonoid contents obtained from the extract. Phenolic compounds are widely recognized for their redox properties, enabling them to neutralize reactive oxygen species through hydrogen atom or electron donation. The strong antioxidant activity observed in the DPPH assay supports this mechanism. Oxidative stress is a key contributor to the pathogenesis of type 2 diabetes mellitus, as it impairs pancreatic β-cell function and promotes insulin resistance. Therefore, the antioxidant capacity of the extract is highly relevant in the context of diabetes management. The very low IC₅₀ value obtained in the DPPH assay indicates a strong ability of the extract to scavenge free radicals. This antioxidant potential is consistent with the high abundance of polyhydroxylated phenolic compounds. Flavonoids and anthocyanins, in particular, are known to stabilize free radicals through resonance structures. The correlation between total phenolic content and antioxidant activity has been widely reported in previous studies. The high proportion of flavonoids within the total phenolic fraction suggests their major contribution to radical scavenging activity. Such antioxidant effects may indirectly support glycemic control by reducing oxidative damage to insulin-sensitive tissues. These findings indicate that the extraction strategy successfully preserved compounds responsible for antioxidant bioactivity. The antioxidant profile of the extract provides a strong foundation for its subsequent antidiabetic evaluation.

Beyond antioxidant activity, the extract demonstrated pronounced antidiabetic potential through inhibition of carbohydrate-digesting enzymes. The α-glucosidase inhibition assay revealed strong activity at low concentrations, indicating high potency for a crude plant extract. In contrast, inhibition of α-amylase required much higher concentrations, reflecting moderate to weak activity. This selective inhibition pattern is considered beneficial, as excessive α-amylase inhibition is often associated with gastrointestinal side effects. Selective α-glucosidase inhibition delays glucose release at the intestinal brush border without severely disrupting starch digestion. Such a mechanism is advantageous for controlling postprandial hyperglycemia. The observed enzyme selectivity suggests that the extract may provide glycemic regulation with improved tolerability. The strong α-glucosidase inhibition can be attributed to the presence of specific phenolic compounds identified in the extract. LC–MS/MS analysis revealed a diverse metabolite profile dominated by flavonoids, anthocyanins, and phenolamides. Compounds such as quercetin, kaempferol, and their glycosides have been reported to inhibit α-glucosidase through interactions with catalytic residues. Anthocyanins are also known to modulate glucose metabolism and suppress postprandial glucose spikes. Phenolamides such as tricoumaroyl spermidine contribute additional antioxidant and enzyme-modulating properties. The coexistence of these compounds suggests a multifactorial mechanism of antidiabetic action. Synergistic effects among phenolics may enhance enzyme inhibition beyond the activity of individual compounds. The weaker α-amylase inhibition observed is consistent with the phytochemical composition, which appears richer in flavonoids than in tannin-type polymers. This enzymatic profile aligns well with current strategies targeting selective carbohydrate digestion. The *in vitro* antidiabetic results therefore reflect both the qualitative and quantitative composition of the extract.

Molecular docking analysis provided structural insights into the interaction mechanisms between identified compounds and target enzymes. Docking simulations demonstrated that several compounds exhibited favorable binding free energies and low predicted inhibition constants against α-glucosidase. Effective ligands formed multiple hydrogen bonds with key catalytic residues, particularly Asp616, Asp404, and Asp282. These residues are essential for proton transfer and substrate cleavage during catalysis. The formation of stable hydrogen-bond networks indicates strong ligand anchoring within the active site. Hydrophobic interactions further contributed to complex stabilization in certain ligands. Compounds that reproduced interaction patterns similar to the native ligand showed enhanced predicted inhibitory potency. Docking against α-amylase revealed generally weaker binding affinities, consistent with the experimental inhibition data. Although some ligands formed strong interactions, effective inhibition required higher concentrations. Hydrogen bonding with residues such as Glu233 and Asp300 played a decisive role in stabilizing ligand–enzyme complexes. Ligands dominated by hydrophobic interactions showed reduced predicted inhibition. The agreement between docking results and *in vitro* enzyme assays strengthens the reliability of the proposed mechanisms. The computational findings support the selective inhibition of α-glucosidase over α-amylase. This selectivity reflects both binding site compatibility and functional group orientation of the ligands. The integration of LC–MS/MS and docking data links chemical composition directly to biological activity. The consistency between experimental and *in silico* results enhances confidence in the identified bioactive compounds. These findings highlight the molecular basis underlying the antidiabetic effects of the extract. The study demonstrates that *Rosa hybrida* flower extract exerts antidiabetic activity through complementary antioxidant and enzyme-inhibitory mechanisms.

## 5 Conclusions

The present study investigated the antidiabetic potential of *Rosa hybrida* Hort. flower extract using integrated *in vitro* and *in silico* approaches. The extract exhibited very strong antioxidant activity, as indicated by an IC₅₀ value of 9.775 µg/mL in the DPPH assay. High levels of total phenolic content (60.508 mg GAE/100 mg extract) and total flavonoid content (38.507 mg QE/100 mg extract) were also observed, supporting its antioxidant capacity. *In vitro* enzyme inhibition assays demonstrated potent α-glucosidase inhibitory activity, with an IC₅₀ value of 4.45 ppm, while the inhibitory effect against α-amylase was moderate to weak, with an IC₅₀ of 17,149.28 ppm. LC–MS/MS profiling identified 22 compounds, predominantly flavonoids, anthocyanins, phenolamides, and terpenoids. Molecular docking simulations further revealed strong binding affinities and favorable interaction patterns of several key compounds with both α-glucosidase and α-amylase. These findings provide scientific evidence supporting the antidiabetic potential of *Rosa hybrida* flower extract, mediated through its antioxidant properties and selective inhibition of carbohydrate-digesting enzymes.

## List Abbreviation

**Abbreviation Full Term**
DM: Diabetes Mellitus
T2DM: Type 2 Diabetes Mellitus
DPPH: 2,2-Diphenyl-1-picrylhydrazyl
TPC: Total Phenolic Content
TFC: Total Flavonoid Content
LC–MS/MS: Liquid Chromatography–Tandem Mass Spectrometry
UHPLC: Ultra-High Performance Liquid Chromatography
Q-TOF: Quadrupole Time-of-Flight
MS/MS: Tandem Mass Spectrometry
RMSD: Root Mean Square Deviation
PLIP: Protein–Ligand Interaction Profiler
VMD: Visual Molecular Dynamics
UV–Vis: Ultraviolet–Visible Spectrophotometry
IC₅₀: Half-Maximal Inhibitory Concentration
GAE: Gallic Acid Equivalents
QE: Quercetin Equivalents
SMILES: Simplified Molecular Input Line Entry System
DNS: 3,5-Dinitrosalicylic Acid
*m/z*: Mass-to-Charge Ratio
eV: Electron Volt

## CRediT authorship contribution statement

**Saeful Amin:** Writing – review & editing, Visualization, Software, Resources, Data curation. **Salsabila Adlina:** Writing – review & editing, Visualization, Software, Resources, Data curation. **Jajang Japar Sodik:** Writing – review & editing, Writing – original draft, Visualization, Validation, Software, Resources, Methodology, Investigation, Formal analysis, Data curation, Conceptualization. **Risa Susanti:** Writing – review & editing, Visualization, Software, Resources, Data curation. **Taufik Muhammad Fakih:** Writing – review & editing, Writing – original draft, Visualization, Validation, Software, Resources, Methodology, Investigation, Formal analysis, Data curation, Conceptualization. **Nabila Hadiah Akbar:** Writing – review & editing, Visualization, Software, Resources, Data curation. **Entris Sutrisno:** Writing – review & editing, Writing – original draft, Validation, Supervision, Project administration, Methodology, Investigation, Funding acquisition, Formal analysis, Conceptualization.

## Declaration of Competing Interest

The authors declare that they have no known competing financial interests or personal relationships that could have appeared to influence the work reported in this paper.

## Data Availability

The authors do not have permission to share data.
